# Fog-Based Smart Cardiovascular Disease Prediction System Powered by Modified Gated Recurrent Unit

**DOI:** 10.3390/diagnostics13122071

**Published:** 2023-06-15

**Authors:** A Angel Nancy, Dakshanamoorthy Ravindran, Durai Raj Vincent, Kathiravan Srinivasan, Chuan-Yu Chang

**Affiliations:** 1 Department of Computer Science, St. Joseph’s College (Autonomous), Bharathidasan University, Tiruchirappalli 620002, India; angelnancy_phdcs@mail.sjctni.edu (A.A.N.); ravindran_cs1@mail.sjctni.edu (D.R.); 2 School of Information Technology and Engineering, Vellore Institute of Technology, Vellore 632014, India; pmvincent@vit.ac.in; 3 School of Computer Science and Engineering, Vellore Institute of Technology, Vellore 632014, India; kathiravan.srinivasan@vit.ac.in; 4 Department of Computer Science and Information Engineering, National Yunlin University of Science and Technology, Douliu City 64002, Taiwan; 5 Service Systems Technology Center, Industrial Technology Research Institute, Hsinchu 310401, Taiwan

**Keywords:** cloud computing, cardiovascular disease, fog computing, Internet of Things, healthcare, heart attack, predictive analytics, recurrent neural network, gated recurrent unit

## Abstract

The ongoing fast-paced technology trend has brought forth ceaseless transformation. In this regard, cloud computing has long proven to be the paramount deliverer of services such as computing power, software, networking, storage, and databases on a pay-per-use basis. The cloud is a big proponent of the internet of things (IoT), furnishing the computation and storage requisite to address internet-of-things applications. With the proliferating IoT devices triggering a continual data upsurge, the cloud–IoT interaction encounters latency, bandwidth, and connectivity restraints. The inclusion of the decentralized and distributed fog computing layer amidst the cloud and IoT layer extends the cloud’s processing, storage, and networking services close to end users. This hierarchical edge–fog–cloud model distributes computation and intelligence, yielding optimal solutions while tackling constraints like massive data volume, latency, delay, and security vulnerability. The healthcare domain, warranting time-critical functionalities, can reap benefits from the cloud–fog–IoT interplay. This research paper propounded a fog-assisted smart healthcare system to diagnose heart or cardiovascular disease. It combined a fuzzy inference system (FIS) with the recurrent neural network model’s variant of the gated recurrent unit (GRU) for pre-processing and predictive analytics tasks. The proposed system showcases substantially improved performance results, with classification accuracy at 99.125%. With major processing of healthcare data analytics happening at the fog layer, it is observed that the proposed work reveals optimized results concerning delays in terms of latency, response time, and jitter, compared to the cloud. Deep learning models are adept at handling sophisticated tasks, particularly predictive analytics. Time-critical healthcare applications reap benefits from deep learning’s exclusive potential to furnish near-perfect results, coupled with the merits of the decentralized fog model, as revealed by the experimental results.

## 1. Introduction

The global revolution brought about by the reign of the digital age has touched all walks of human life and ushered in expeditious changes that are reshaping economies and societies and opening up immense possibilities while transforming lives. Given this, the centralized cloud computing model offers ubiquitous access to resources required for processing, analytics, and storage in a conducive manner [1,2]. The internet of-things (IoT), which seamlessly connects objects and people, has triggered a data upsurge due to the rapidly expanding number of connected devices. The data thus acquired warrants analysis, identifying patterns and trends to infer insights for efficient decision-making. However, IoT devices possess minimal computational and storage potential. These limitations are overcome by integrating cloud computing with IoT, termed the Cloud-of-Things [3]. In that context, the cloud has become the key enabler of IoT, offering services across healthcare, education, and industry domains while overcoming significant IoT restraints.

The World Health Organization (WHO) states that cardiovascular diseases claim 17.9 million lives globally annually [4]. Healthcare applications deploy artificial intelligence, specifically machine learning techniques, to elicit intelligent insights [5] from the exponentially increasing medical data. These clinical prediction models aid in diagnosing heart disease and prognosis by accurately predicting future medical events, thereby improving individuals’ health [6]. The proliferating number of healthcare internet-of-things devices, such as smart wearable ones, generates massive amounts of data, transmitted to the cloud for computation. The cloud possesses the improved processing power these machine learning models warrant [7]. The data transfer between these healthcare IoT devices and the cloud servers requires considerable bandwidth. Furthermore, it increases latency, which is inept for real-time healthcare applications that are, by and large, latency-sensitive [8]. The fog computing paradigm, put forth by Cisco, positions the fog layer closer to the IoT layer, where data is produced, and conveniently extends the cloud services to the edge [9]. The inclusion of an additional layer with computational capability amid the cloud layer and the IoT layer considerably minimizes the response time, making inpatient care and remote monitoring possible. With healthcare applications warranting swift reaction times, fog computing is a viable alternative to managing the complexity of vast medical data, eventually enhancing reliability [10]. Thus, this assures that the cloud is reserved for large-scale, complex analytics tasks. The potential to triage data while eliciting vital decisions within the device’s environment, as in fog, will help extract critical insights from the vast volume of data [11].

### 1.1. Motivation

The cloud servers traditionally manage the pre-processing, analysis, and storage of the enormous IoT data generated at varying velocities from sizable IoTs. These are eventually sent to the cloud datacenters, and the computation result is returned to the IoT devices, leading to transmission and processing errors alongside needless delays [12]. The high latency in handling real-time medical data may render it inadequate, meaningless, and unreliable. With expanding data sizes, delays due to round-trip time may increase from milliseconds to minutes, adversely impacting the quality of service (QoS) of healthcare IoTs’ real-time operations. The crucial necessities of healthcare IoTs include reducing latency and conserving network bandwidth. IoT devices are linked to the cloud by several routers and gateways, causing data to travel a long distance and consume high bandwidth, causing needless delays [13]. An intensive care unit (ICU) patient or a home-bound cardiac patient under monitoring warrants swift actions as soon as a drop in vital signs is detected, and caretakers are to be promptly notified to avoid it becoming disastrous and fatal [14]. The physiological state of patients varies with time, and remote patient monitoring insists on rapid responses and agile decisions. Thus, most healthcare IoT applications require processing close to IoT devices and hardly need cloud-scale storage and processing.

The huge amount of data amassed by healthcare IoT devices makes it challenging to infer intuitive and streamlined decisions from the electronic clinical records to enhance clinical care’s reliability. With devices becoming tinier and data becoming more sizeable, finding meaningful methods to acquire, analyse, interpret, and use data that can substantially impact patient care is becoming a hassle. With the cloud unable to meet healthcare IoT demands due to its inherent limitations, the fog computing (FC) concept has garnered attention lately. It eases the burden of the centralized cloud by extending its features to the network edge, which can be a router, gateway, or any device mediating the cloud and IoT. With proximity to IoT end devices, FC’s key intent is to mitigate the high latency between the cloud and IoT devices. The healthcare application entails a smart infrastructure that acquires the massive IoT data in real time, performs preliminary computation tasks while reducing network latency and transmission issues, and sends the processing results to the cloud and IoT end nodes. The primary motivation of this research is to enhance decision-making accuracy when handling vast amounts of data, which deep learning models are competent at handling. The research endeavour also emphasizes the minimum latency requisite for time-critical healthcare applications using fog computing technology for enhanced quality of service.

### 1.2. Contributions of This Work

The key outcomes of the research primarily oriented toward diagnosing heart disease risk severity are as follows:

- The smart and efficient heart disease diagnostic system encompasses the IoT–fog–cloud technologies;
- The healthcare IoT data acquired is pre-processed by a filtering technique and fuzzy inference system and subjected to predictive analytics at the fog layer using deep learning’s recurrent neural network model of the gated recurrent unit (GRU);
- The proposed fuzzy inference system with improved GRU accurately predicts heart attack risk from IoT patient data and electronic health records (EHR) when compared to the results of the generic GRU model;
- The suggested model is evaluated using metrics that test the deep learning model’s predictive adeptness and performance, with a comparison of cloud and fog.

The rest of the article is organized into sections on related work, background, proposed methodology, experimental setup, performance evaluation, results and discussion, and conclusions.

## 2. Literature Review

Smart healthcare systems are becoming prevalent and have been revolutionised by merging cloud computing and IoT-based sensor technology. Minor errors in heart disease diagnosis may prove fatal to patients; therefore, ML techniques have been deployed to predict various cardiovascular diseases effectively, thereby minimizing the death ratio [15]. The University of California, Irvine (UCI) Machine Learning Repository’s Heart Disease dataset has been harnessed by many researchers to examine their heart disease classification model’s efficiency, which is discussed as follows [4,16,17,18,19,20,21,22,23,24,25,26,27,28,29]. A study conducted by Singh and Kumar to analyse the effectiveness in detecting heart diseases among the machine learning algorithms of support vector machine, decision tree, k-nearest neighbour, and linear regression concluded that the k-nearest neighbour is the preferred model, with an accuracy of 87% [16]. A similar study by Rajdhan et al., with the same dataset involving a decision tree, naive Bayes, logistic regression, and random forest algorithms, indicates that the best accuracy, of 90.16%, was shown by the random forest algorithm [17]. An Android application based on the cloud was proposed to predict heart disease with algorithms such as naive Bayes, simple logistic regression, random forest, support vector machine, and artificial neural networks, among which support vector machine exhibited the highest accuracy at 97.53% [18]. A heart disease diagnosis system involving two subsystems of relief and rough set feature selection and an ensemble classifier model has shown 92.59% accuracy when evaluated [19]. Classifying heart diseases from electrocardiogram (ECG) signals by using classification and pre-processed methods displays an accuracy of 98.4% when symbolic aggregate approximation for pre-processing and long short-term memory (LSTM) for classification are used [20]. A smart system to ascertain key biomedical markers named BioLearner is proposed for predicting heart disease utilizing machine learning algorithms such as a k-nearest neighbour, neural networks, and support vector machine displays an accuracy of 95% [21]. Predicting heart disease by an ensemble deep dynamic algorithm using linear regression and a deep Boltzmann machine displays an accuracy of 98.12% [22].

In recent times, IoT–fog–cloud-based models for predictive analytics have become prominent owing to the benefits they offer. Fog computing is equipped to proficiently address computational tasks involving healthcare data originating from diverse IoT devices, such as wearable sensors, and prior electronic clinical data stored in the cloud [23]. In particular, fog computing’s operational capability minimizes response time, latency, and delay, which is adept for heart disease patients’ healthcare monitoring. An IoT–fog computing integrated healthcare system called HealthFog has been proposed for heart disease diagnosis [24], which employs ensemble deep learning models of bagging and boosting. Furthermore, it harnesses the FogBus framework [25], which enables IoT–fog–cloud integration and has shown improved accuracy, latency, network bandwidth, jitter, execution time, and power consumption. It is customizable for different operation modes, offering the best prediction accuracy and quality of service. A novel hybrid IoT–fog assisted healthcare model [26] for handling heart disease data based on fuzzy reinforcement learning and neural network evolution strategies at fog nodes has been proffered. It indicates optimal performance results regarding latency, RAM consumption, and network usage under simulation compared to existing techniques.

HealthCloud, a cloud-based monitoring system to identify heart disease risk involving a support vector machine, logistic regression, gradient boosting trees, k-nearest neighbour, and neural networks, unveiled logistic regression as being 85.96% more accurate and responsive [27]. Here, performance was evaluated using the metrics of accuracy, recall, specificity, latency, execution time, and memory usage. An IoT–fog–cloud-based heart disease diagnostic model deploying machine learning algorithms has been put forth, which shows an accuracy of 97.32%, precision of 97.16%, recall of 97.58%, specificity of 96.87%, and F1-measure of 97.37% [28]. A modified deep convolutional neural network model involving smart healthcare monitoring to predict heart disease in the IoT–cloud framework while harnessing the UCI dataset shows an accuracy of 93.3% [29]. An IoT smart system for predicting heart disease with kernel discriminant analysis and a modified self-adaptive Bayesian algorithm displays an accuracy of 90% [30]. An IoT-cloud-based smart heart disease prediction system was suggested to acquire healthcare data from the IoT and utilize a fuzzy inference system for data pre-processing and bidirectional long short-term memory for classification, and showed 98.86% accuracy [4].

Machine learning is deployed not only for heart disease prediction systems but also for other healthcare diagnostic applications. A system is presented to identify the hypertensive stage, wherein IoT sensors acquire the user’s health parameters in real time at the fog layer [31]. An alert is generated once hypertension is identified, and the artificial neural network predicts a hypertension attack risk state in users at remote sites. The results are stored in the cloud, while the temporal information produced at the fog layer can be used to enforce preventive measures for patients’ wellness. This model displays high prediction accuracy, low response time, and bandwidth efficiency. For monitoring, prevention, and control of encephalitis [32], a fog-assisted healthcare model deploying a fuzzy C-Means classifier and a temporal recurrent neural network has shown improved classification and prediction accuracy while minimizing latency and response time. In the research work for a fog-based diabetes patient support system [33], the J48Graft decision tree has been deployed. A fog-based latency-aware framework to monitor and detect dengue viruses involving cloud computing and IoT has been shown to classify users by symptoms with improved execution and response time [34].

## 3. Materials and Methods

### 3.1. IoT–Fog–Cloud Interplay

By 2025, the number of smart, connected devices is forecasted to exceed 75.44 billion, up from 42.62 billion in 2022 [35]. As per the International Data Corporation (IDC), the global data volume produced by IoT devices is predicted to exceed 79.4 zettabytes (ZB) by 2025 [36]. The surging data from IoT-connected devices within the conventional cloud computing model has triggered the cloud’s inherent shortcomings to become apparent as services are orchestrated by distant cloud datacenters [37]. It is inept at managing time-sensitive real-time applications that warrant swifter decision-making and has become insufficient as a myriad of devices accesses the cloud. An efficient quality of service (QoS) assurance is vital for latency-sensitive real-time applications.

The fog computing concept has evolved to be a reliable and resilient extension of the cloud, favourably positioned in between the cloud layer and the IoT device layer [38]. Its proximity to the end users is favourable for efficiently managing computation and communication tasks alongside crucial local resource-sharing and storage-related activities. Fog computing encompasses a geographically distributed architecture involving heterogeneous edge-level devices that provide flexible computation, storage, and communication services [39]. Thus, fog computing complements cloud computing technology by extending it to the network edge, accelerating lightweight local processing of data and responses to events, thereby reducing latency [40]. The data transmission rate is significantly reduced as a round trip to the cloud is mitigated. The increased network bandwidth utilization caused by data traffic to and from the core cloud network is drastically reduced due to local data processing. Unlike the cloud, which possesses unlimited, high-performance processing and storage capability, fog computing has limited resources. With localized analysis, overall business agility and service levels are improved.

### 3.2. Predictive Analytics in Healthcare

Predictive analytics is a field of data analytics involving engineering, scientific, mathematical, and statistical models and data mining techniques, as well as AI and machine learning methods, for foreseeing patterns to make accurate and meaningful decisions [41]. The past and real-time data are used to predict the future. The interest in predictive analytics in healthcare in the recent past is attributed to the availability of flexible and powerful machine learning algorithms and computing platforms alongside high-quality data; more specifically, electronic health record (EHR) data [42].

Accurate, real-time insights based on abundant clinical data that can impact patients are crucial in aiding healthcare organizations, hospitals, and clinicians [43]. Predictive analytics in healthcare analyses past records and current clinical data. It offers clinical decision support for doctors to render a fast and accurate diagnosis, identify at-risk patients, prevent diseases, and provide quality patient care [44]. It further includes identifying deteriorating patient signs in the ICU, remotely providing predictive care to high-risk patients, preventative care for chronic diseases, and detecting the equipment’s maintenance needs. It helps predict where, when, and how to improve health outcomes through enhanced patient care. Healthcare analytics enables averting medical risks rather than treating health problems and empowers clinicians to make informed decisions with patients’ lives at stake [45]. It is a convenient tool that enhances the value of healthcare by being helpful in diagnosis, prognosis, and treatment while saving time and reducing costs.

Traditional statistical methods as well as advanced artificial and machine learning approaches are leveraged, referred to as clinical prediction models. They enable eliciting accurate predictions and identifying the patient’s risk condition from enormous data in less time [46]. Predictive analytics is already becoming a keystone of personalized healthcare treatment and helps warn clinicians and caretakers about the potential of occurrences and outcomes before they happen. This decreases the administrative burden of healthcare professionals and helps tackle administrative and operational challenges. Driven by the widespread deployment of artificial intelligence and the internet of things (IoT), algorithms are fed historical as well as real-time healthcare data to make meaningful predictions on the probability of events to prevent and cure health issues.

### 3.3. Recurrent Neural Network (RNN)

All domains encompassing industry, healthcare, agriculture, education, banking, and finance are, without exception, permeated by machine learning (ML) techniques. Explicitly, deep learning (DL), which attempts to mimic the human brain’s functionality, has been garnering significance recently as a subfield of machine learning [47]. It can learn from an enormous amount of data and, with additional hidden layers, optimise and refine predictions with incredible accuracy. DL drives many everyday AI products and services that perform without human intervention. Deep learning has proven it is able to solve intricate predicaments as a supervised learning model, with notable breakthroughs in computer vision, natural language processing, autonomous vehicles, fraud detection, and healthcare. Certain deep learning neural networks or artificial neural networks are profoundly sophisticated, addressing specific cases and datasets [48]. Convolutional neural networks (CNNs) have efficiently handled computer vision, image pattern recognition, and classification tasks [49]. Recurrent neural networks (RNNs) leverage sequential or time series data in natural language processing, speech recognition, meteorological data, and predictive healthcare [50].

### 3.4. Gated Recurrent Unit (GRU)

In addition to manually recorded unstructured data, the surge in multi-sensor systems has led to episodic time series data. The conventional deep learning techniques fail due to the irregularities and incompleteness of time series data [51]. From our foray into deep learning (DL), we can report that recurrent neural networks (RNNs) are worth investigating owing to their state-of-the-art solutions in modelling time series data. They compute each time step’s hidden state using the previous hidden state and the current input through a weight matrix and activation function, enabling retention of information from past inputs. In spite of the standard RNN’s adeptness at handling temporal dependencies, it suffers from vanishing gradient problems. This problem arises when the backpropagation gradients become too small to update the network parameters, thereby impairing the network’s ability to learn long-term dependencies. However, RNN’s variants of long short-term memory (LSTM) and gated recurrent unit (GRU) have shown phenomenal results in sequential modelling [50,52]. It is an apparent choice for handling irregular as well as time series data. Hence, a GRU is worth a try in predictive healthcare. It is designed to overcome the vanishing gradient issue and efficiently capture long-term dependencies by incorporating gated mechanisms that streamline the network’s information flow.

**Figure 1:**
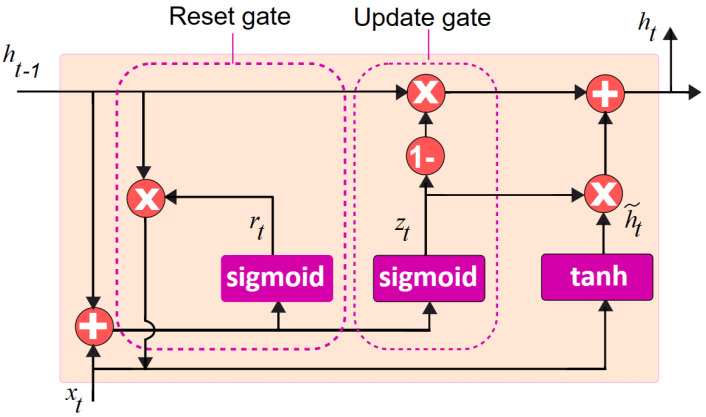
Standard gated recurrent unit model.

GRU is a gated RNN, propounded by Kyunghyun Cho et al., that employs the reset and update gates to regulate selective retention or forgetting of information from prior time steps [53]. At each time step, the GRU acquires an input vector and a hidden state vector to generate an output vector and a new hidden state vector. The hidden state basically includes a value ‘1’ or ‘0’ indicating that the previous hidden state is fully retained or forgotten, respectively. The update gate helps determine the past information that is to be retained and passed on to the current hidden state from the previous hidden state and current input. The reset gate decides which past knowledge to forget from the previous hidden state and how much of the current input to focus on [54]. Thus, the GRU model learns through training by backpropagation involving weight matrices and activation functions. The activation function sigmoid is used for the update and reset gate. The tanh activation function is used to compute the current hidden state. GRU is much simpler, as it includes fewer parameters, making it easier and faster to train while consuming less memory [55]. The gated recurrent unit can be referred to as memory-centred, as it is capable of retaining information over a while. The equations of a standard GRU are delineated as follows:

(1) $$\mathrm{Update}\;\mathrm{gate}:\;z_{t}=\sigma \left(W_{z}x_{t}+W_{z}h_{t-1}\right)$$

(2) $$\mathrm{Reset}\;\mathrm{gate}:\;r_{t}=\sigma \left(W_{r}x_{t}+W_{r}h_{t-1}\right)$$

(3) $$\mathrm{Candidate}\;\mathrm{hidden}\;\mathrm{state}:\;\tilde{h_{t}}=\tanh\left(Wx_{t}+Wr_{t}+Wh_{t-1}\right)$$

(4) $$\mathrm{Hidden}\;\mathrm{state}:\;h_{t}=\left({1-z}_{t}\right)*h_{t-1}+z_{t}*\tilde{h_{t}}$$

where *z*, *r* are the update and reset gates, respectively, while *W*(*z*,*r*) corresponds to their weight matrices. $\tilde{h_{t}}$ and $h_{t}$ indicate at time *t* the linear interpolation among the current $\tilde{h_{t}}$ and previous $h_{t}$ activation states, while $x_{t}$ refers to the input. σ and tanh are the sigmoid and the hyperbolic tangent activation functions, respectively. The element-wise multiplication is indicated by the ∗ operator. Figure 1 depicts the standard gated recurrent unit model [56].

In Figure 1, $x_{t}$ is the input at time step *t*, $h_{t-1}$ denotes the hidden state of the previous time step, and sigmoid and tanh are the activation functions. The update gate $z_{t}$ determines how much information to withhold from the previous hidden state. The reset gate $r_{t}$ decides how much of the new input should be considered. The candidate hidden state $h_{t}$ is the intended new hidden state, which is the combination of the input and the reset gate. The actual hidden state, $h_{t},$ is based on both the candidate hidden state as well as the previous hidden state. The output is generated based on the hidden state $h_{t}$.

### 3.5. Proposed Model

IoT-connected devices acquire data for real-time healthcare applications. With surging IoT devices comes the challenge of managing the computation and storage of an exorbitant volume of medical data generated from the connected devices.

**Figure 2:**
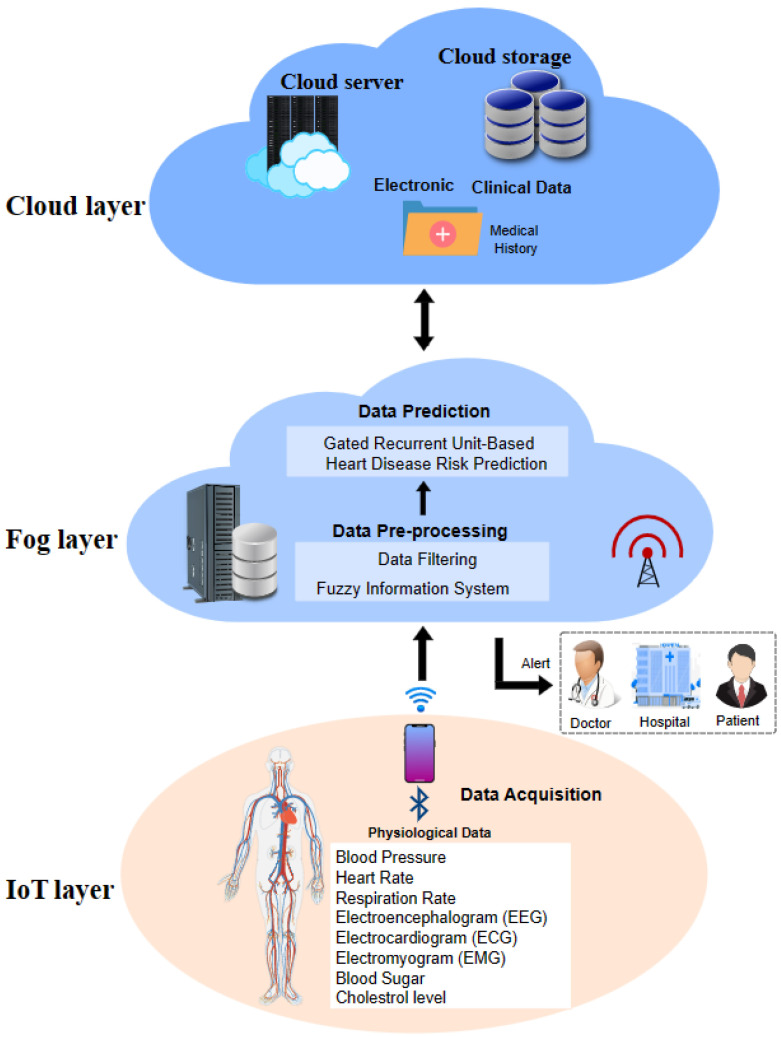
Smart system for heart disease risk prediction.

The propounded smart heart disease risk prediction system, as portrayed in Figure 2, comprises modules of (1) Data collection, (2) Data pre-processing, and (3) Disease risk prediction.

#### 3.5.1. Data Collection Layer

The proposed predictive smart healthcare system has two main data sources: physiological data acquired from remote health monitoring smart devices, and electronic health records (EHR). The data gathered from routine monitoring of patients using smart connected devices include blood pressure (BP), blood sugar level, respiration rate, heart rate, blood oxygen level, cholesterol level, electrocardiogram (ECG), electroencephalogram (EEG), and electromyogram (EMG). The electronic clinical data include an extensive medical history comprising lab reports, observations, medications, and allergies that offer valuable disease prediction information, which is stored in the cloud database. The acquired data is sent to associated fog layer nodes, such as gateway devices, for further analysis, via wireless technologies, Bluetooth, or Zigbee [57], which enable interaction between IoT devices.

- Dataset

The heart disease datasets from the Hungarian Institute of Cardiology and Cleveland Clinic Foundation were accessed from the UCI machine learning repository [58]. This fairly balanced dataset collectively had 597 records with 76 actual attributes, of which 13 features were selected for this research to detect heart attack risk in patients. Table 1 depicts the characteristics of the dataset considered.

Table 2 depicts the sample of the dataset considered.

#### 3.5.2. Data Pre-Processing Layer

With real-world data likely to be incomplete, inconsistent, and noisy, data pre-processing prior to ML model deployment has become inevitable. The wearable sensor data is prone to noise and missing data owing to signal aberrations, which may impact the accuracy of the heart disease prediction system. Therefore, the Kalman filtering technique, which operates with minimal computing power, is used to remove noise, duplicate records, and discrepancies in data of concern [59,60]. This technique is apt to manage huge amounts of sensor data and can deliver values near actual sensor data while eliminating noise.

The initial classification of heart disease risk based on the patient’s health data, namely blood pressure, maximum heart rate, and ECG, is done using the fuzzy inference system (FIS) [4]. The fuzzy system is used to model real-world occurrences that are innately vague and derived from the logical decisions made by humans using imprecise as well as non-numerical inferences. Fuzzy inference maps input to output based on fuzzy logic to ensure discernment of patterns for accurate decision-making. In the proposed system, the values of maximum heart rate, ECG, and blood pressure and their respective member functions served as input. The member functions taken for the three inputs were low, normal, and high. They were then fuzzified using a fuzzy value range into fuzzy sets. As per the classification results, high-risk patients and their overall status were stored for future analysis on the cloud server. The high-risk patient data was further analysed at the subsequent prediction layer. The algorithm for a fuzzy inference system (FIS) for classifying the risk of heart disease using blood pressure, ECG, and maximum heart rate is illustrated in Algorithm 1.
**Algorithm 1:** Classifying patients by their health data by FISStep 1: Inputs and relevant member functions define the fuzzy systemStep 2: Calculate the risk of heart disease by µ_1_(ECG_1), µ_1_(MaxHeartRate_1), µ_1_(BloodPressure_1) as µ_1_(high) or µ_1_(normal) or µ_1_(low)Step 3: If HealthRiskState = µ_1_(high)   3.1 Notify G_D_ using SPARK as RTA   3.2 Store P_uid_ in F_S_ and C_S_Step 4: Otherwise store P_uid_ HealthRiskState in C_S_Step 5: Stop the process

G_D_: Gateway device; C_S_: Cloud server; F_S_: Fog server; µ_1_: Membership function; RTA: Real-time analyser; P_uid_: Patient unique identification number

Note: SPARK is the open-source analytical engine issuing real-time analytics.

An alert is sent regarding patients found to be at high risk for heart disease, and the health status results are stored in the cloud for future reference.

#### 3.5.3. Data Prediction Layer

After the initial pre-processing tasks were completed, the patients classified as being at high risk for cardiac disease were additionally subjected to intense analysis at the subsequent prediction layer. This research initiative modifies the RNN’s variant of GRU for the prediction task. The modified GRU model classified cardiac disease states accurately as having risk or not and was found to outperform the standard GRU’s prediction accuracy. The performance-enhanced GRU model description and the ensuing performance evaluation are delineated as follows:

##### Modified GRU Model

The gated recurrent unit (GRU) is the latest advancement of the standard recurrent neural network model intended to overcome the vanishing gradient impediment coupled with faster computational capability and less memory consumption [53,54,56,61]. If a layer fails to learn in a neural network, the RNNs forget longer sequences. The GRU solves this issue by deploying two gates—the update and reset gates—that determine whether the information is allowed through to the output, and it retains information from farther back, respectively. Thus, it enables passing only relevant information from a sequence of events for better predictions. The update gate updates the weights and helps eliminate the problem of vanishing gradient. The model learns on its own and continues to update the information to pass on to the future. In contrast, the reset gate determines the information that needs to be forgotten concerning the current state.

**Figure 3:**
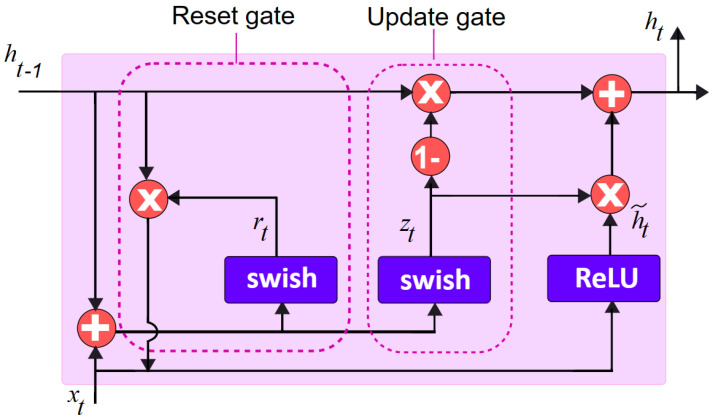
Modified gated recurrent unit model.

The choice of the activation function is found to have a significant influence on the training dynamics as well as the task performance in deep neural networks. Hence, the swish activation function propounded by the Google Brain Team [62] is chosen for the predicted model, which is given as $f_{x}=x.sigmoid\left(\beta x\right)$. Figure 3 shows the modified GRU model.

(5) $$z_{t}=swish\left(W_{z}x_{t}+W_{z}h_{t-1}\right)$$

(6) $$r_{t}=swish\left(W_{r}x_{t}+W_{ri}h_{t-1}\right)$$

(7) $$\tilde{h_{t}}=\mathrm{ReLU}\left(Wx_{t}+Wr_{t}+Wh_{t-1}\right)$$

(8) $$h_{t}=\left({1-z}_{t}\right)*h_{t-1}+z_{t}*\tilde{h_{t}}$$

where *z*, *r* are the update and reset gates, respectively, while *W*(*z*,*r*) corresponds to their weight matrices, and $\tilde{h_{t}}$ and $h_{t}$ indicate, at time *t*, the linear interpolation among the current $\tilde{h_{t}}$ and previous $h_{t}$ activation states. The activation functions in the update and reset gates in the standard gated recurrent unit are sigmoid $\left(\sigma \right),and$ are depicted in Equations (1) and (2). These are replaced by the swish activation function, in the proposed gated recurrent unit model which is indicated in Equations (5) and (6). Similarly, the current memory gate, $\tilde{h_{t}}$, of the standard GRU having the tanh activation function stated in Equation (3) is replaced by the rectified linear unit (ReLU) activation function as depicted in Equation (7) in the propounded model.

##### Experimental Setup

The experiment assesses the deep learning models’ abilities, namely the standard GRU, the combined FIS-GRU (FGRU), and the suggested model, by harnessing the Hungarian and Cleveland heart disease dataset, from the University of California, Irvine (UCI) online ML and data mining repository [58]. This dataset collectively had 597 records with 14 features. The records were synthetically increased using the dataset generator tool, Mockaroo, to 100,000 records to assess the robustness of the proposed model. The IoT data gathered from smart health monitoring devices pertaining to heart disease risk were transmitted to fog devices, wherein the pre-processing and classification took place. Once the pre-processing data tasks were dealt with, the resulting patient data was subjected to three distinct deep prediction models. These comprised the generic GRU model; the fuzzy inference system (FIS) combined with the GRU, indicated by FGRU, wherein the initial classification was performed by the FIS, followed by the GRU for the final prediction; and the proposed GRU model, which also included FIS for the initial classification task.

For the experiment, a system with Intel(R) Core i5-10210U CPU @ 2.11 GHz, 8.00 GB RAM, and 64-bit Windows 10 connected to the intermediary i2k2 cloud infrastructure to rent the Amazon cloud server. The number of fog nodes used ranged from 100 to 1000, with CPU @ 12–80 MHz, 16 KB to 1 MB RAM, 4 MB to 32 MB storage, and IEEE 802.11 Wi-Fi. In addition, the Google Brain team’s open-source machine learning framework of TensorFlow [63] was also utilized. The Apache Spark server, a scalable, in-memory, and open-source framework that has surfaced as the de facto leader among machine-learning-based analytics having faster memory access and disk access, was utilized as well [64,65]. Furthermore, the open-source Apache Cassandra, offering distributed storage infrastructure, was used for this research [66]. The past medical data of patients was accessed from the cloud, and it also stored the final results for future analysis. The suggested neural network model based on the gated recurrent unit consisted of four layers, of which two were hidden. There were five units in the dense layer, and the dropout rate ws 16%. The random weight initialization ranged from 0.1 to 0.2. The decay and learning rates were 0.94 and 0.018, respectively. The optimizer used was Adam; the number of epochs was set to 765 and the batch size was 128. Table 3 indicates the hyperparameter values of the proposed model.

##### Performance Evaluation

For training and testing tasks, the 100,000 patient medical records were partitioned into 70% and 30%, respectively, as this offered the highest accuracy and goodness of fit [67]. The entire dataset of 100,000 records was validated by k-fold cross-validation, wherein the dataset was shuffled and split into k groups. Here, k = 10 was chosen, so the records were divided into ten equal-size sub-samples of 10,000 records. Thus, k − 1, which was 9 folds, was used for training, and the one holdout fold was used for testing. Tuning the neural network model’s hyperparameters and then testing it with entirely unseen data helped prevent overfitting; hence, the cross-validation technique was chosen. After repeating this procedure for all sub-samples, the models were evaluated for accuracy, precision, sensitivity, specificity, and the F1 measure for classifying the heart disease risk status.

##### Evaluation Metrics

The deep learning models’ efficiency was evaluated by the measures of accuracy, precision, recall, specificity, and F1-score [68,69]. The overall predictive ability of the model was revealed by the accuracy metric, which compared the desired output with the actual output. The classification model’s ability to identify heart disease risk in a patient was examined by the values of true positive (TP) and true negative (TN). False positives (FP) and false negatives (FN) pointed out the wrong predictions made by the model. The proportion of actual positive observations to all positive observations was determined by precision. Recall and specificity were the proportions of overall positive and negative observations, respectively. F1-measure was the mean of recall and precision.

## 4. Results and Discussion

The comparison of the generic GRU model; fuzzy inference system (FIS) combined with GRU, indicated by FGRU; and the proposed GRU model was performed, and results in terms of accuracy, precision, recall, specificity, and F1-score were analysed. The three deep models of concern were evaluated by increasing the records from 10% to 100%. The results of a comparative analysis vividly proved that the proposed smart heart attack risk prediction system involving a fuzzy inference system for preliminary analysis and a modified gated recurrent unit for predictive analytics performed better.

**Figure 4:**
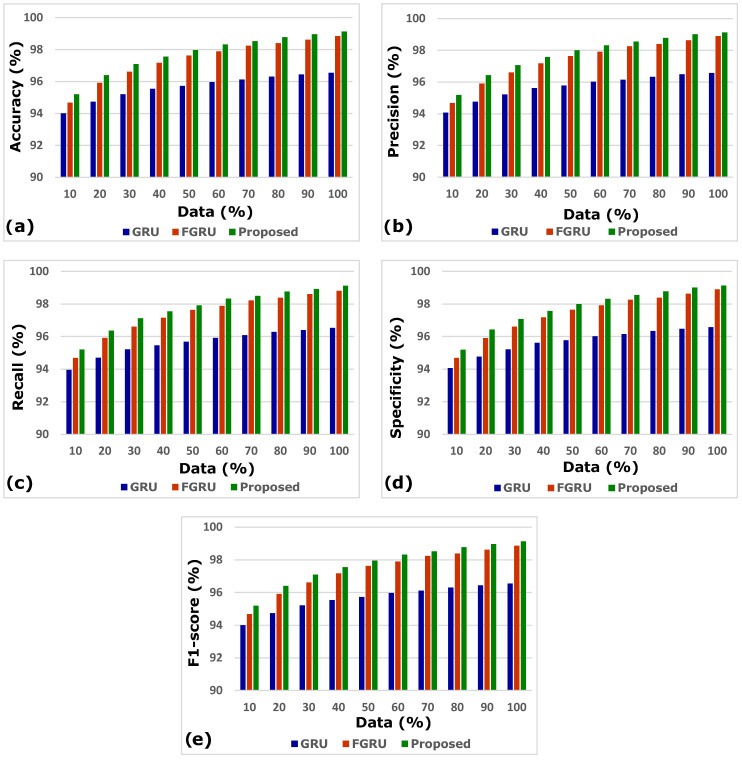
(**a**–**e**) Accuracy, precision, recall, specificity, and F1-score analysis.

Figure 4a–e depicts the accuracy, precision, recall, specificity, and F1-score analysis of the models GRU, FGRU, and the proposed system. The overall performance measures of the GRU, FGRU, and proposed model are hereby compared in Table 4.

**Figure 5:**
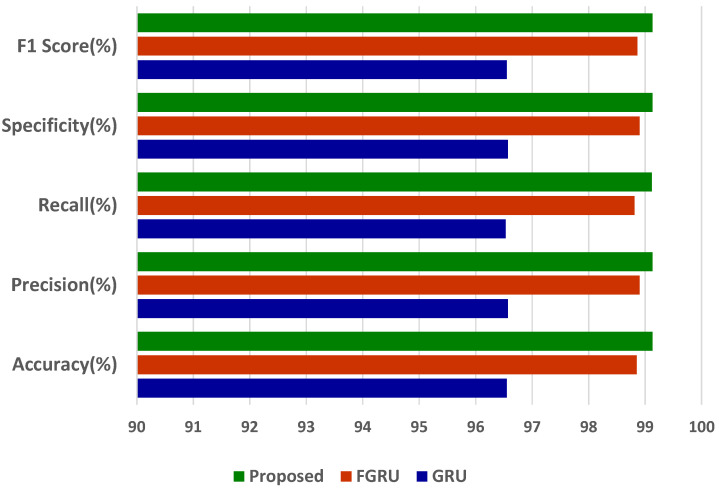
Overall performance results of the proposed model.

The results of accuracy, precision, recall, specificity, and F1-score analysis indicate that the propounded model outperforms the other models of GRU and FGRU. Figure 5 portrays the proposed model’s comprehensive performance.

The proffered model has outdone the other models with an accuracy of 99.13%, a precision of 99.13%, a sensitivity of 99.12%, a specificity of 99.13%, and an F1-score of 99.13%. From the accuracy of 99.13%, we infer the misclassification or error rate (e) using the following formula,

(9) $$Miscalssification\;rate\;(or)\;Error\;rate=\frac{\left(FP+FN\right)}{Total}$$

gives the misclassified rate of disease prediction, which is equivalent to

(10) Misclassification rate or Error rate=1−Accuracy

Thus, the error rate of the proposed healthcare system for disease prediction is 0.0087.

**Figure 6:**
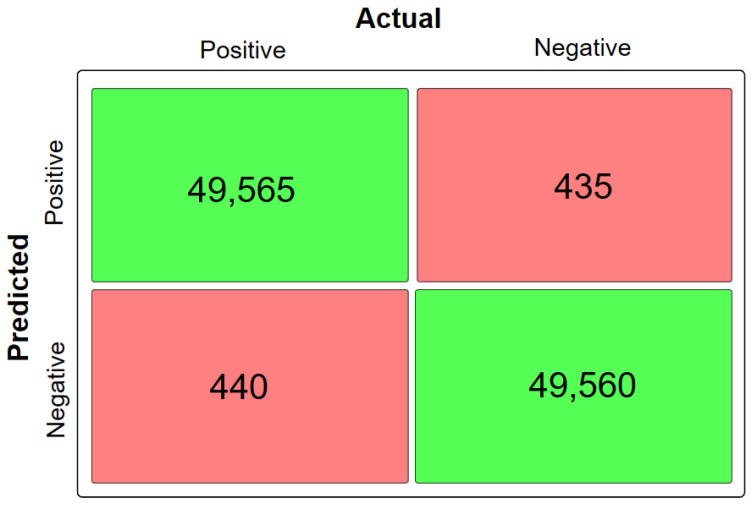
Confusion matrix of the proposed model.

Moreover, the model was evaluated for its efficiency using the confusion matrix depicted in Figure 6.

**Figure 7:**
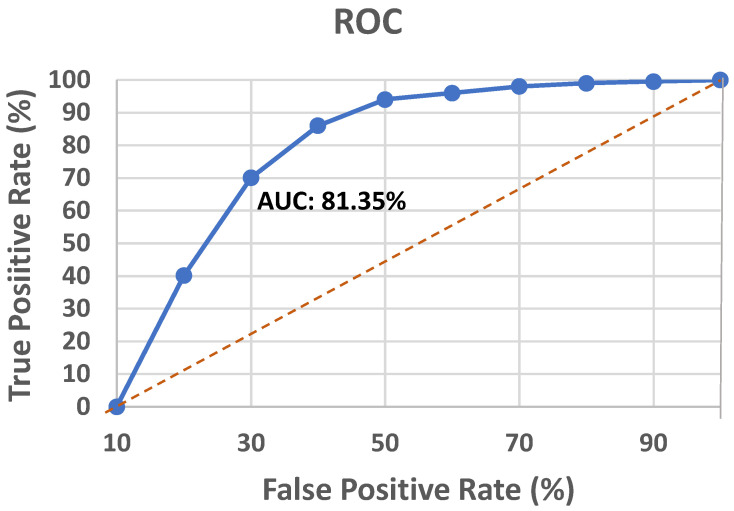
ROC analysis of the proposed model.

The receiver operating characteristics (ROC) curve is a classification model’s key performance indicator that is plotted using the true positive rate (TPR) and false positive rate (FPR) at different classification thresholds. For binary classification problems with an unbalanced dataset, the area under the ROC curve (AUC) score is preferred to the accuracy measure. Figure 7 portrays the ROC curve for the proposed approach.

The AUC score of the model was found to be 81.35%. However, the UCI heart disease dataset harnessed to evaluate the propounded model is reasonably balanced, so the accuracy score supersedes the AUC score.

Mean average precision (mAP) is another common metric used to ascertain a classification model’s performance. It is computed based on the average precision value derived from precision and recall. The mAP of the model was found to be 98.43%.

### 4.1. Comparison with State-of-the-Art Systems

The classification accuracy of the state-of-the-art models harnessing the UCI heart disease dataset was compared with the proposed work. The comparative analysis with the existing literature is characterized by increasing order of accuracy in Table 5.

### 4.2. Comparative Analysis—Fog vs. Cloud

**Figure 8:**
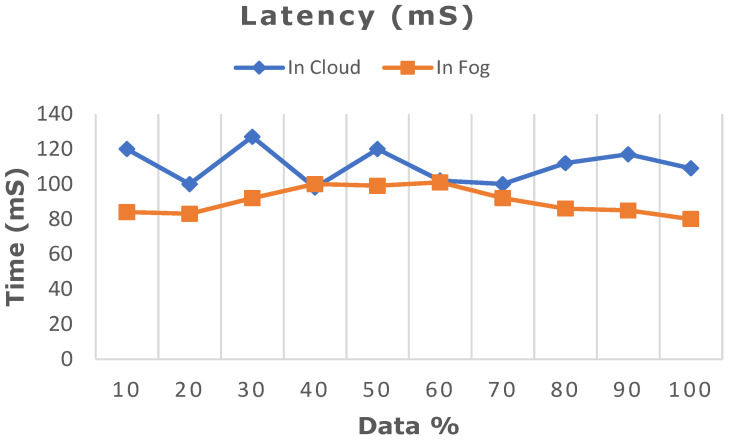
Comparative analysis of latency rate between cloud and fog computing.

**Figure 9:**
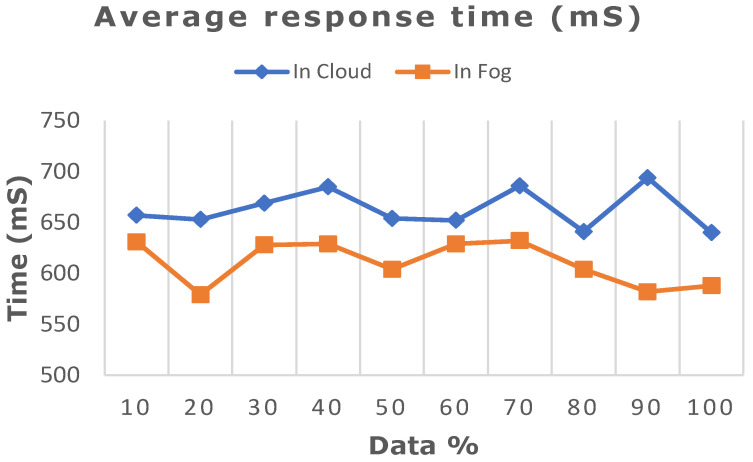
Comparative analysis of average response time between cloud and fog computing.

**Figure 10:**
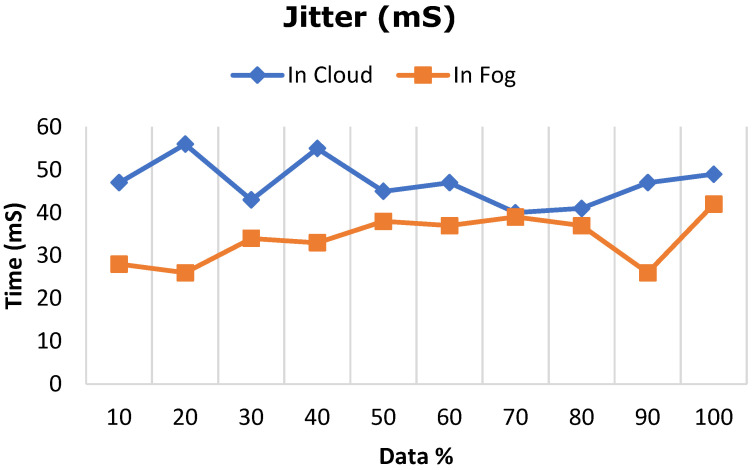
Comparative analysis of network jitter between cloud and fog computing.

**Figure 11:**
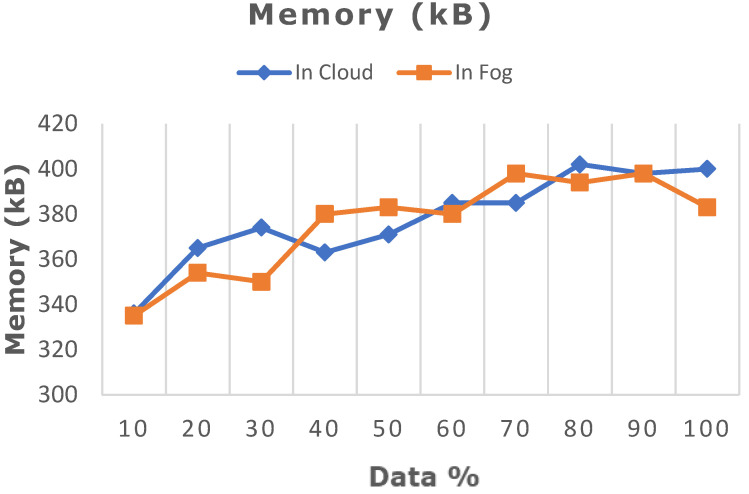
Comparative analysis of memory utilization between cloud and fog computing.

The deep learning model proposed in this work was ascertained by increasing the dataset from 10% to 100% in both cloud- and fog-based computing. The model’s performance in terms of latency rate, jitter, average response time, and memory utilization was evaluated in cloud and fog. Figure 8 depicts the evaluated network latency results. It indicates that classifying the patient’s risk for heart disease by the proposed gated recurrent model involving the fog layer showed less latency relative to that of cloud-based computing. The transmission delay effectuated due to network congestion was referred to as jitter and was examined in cloud and fog, and the results in terms of time are indicated in Figure 9. The network jitter in the fog was comparatively lower than that in the cloud. The average response time in fog and cloud was also evaluated, and the results in fog showed a lower response time in the fog than in the cloud, which is depicted in Figure 10. Moreover, the memory utilization of the proposed classification model was ascertained, with fog showing better results due to offloading to nearer fog nodes than the faraway cloud, and the results are portrayed in Figure 11. With fog computing offering processing as well as communication resources in proximity to the end users, it considerably minimizes data transmission traffic and leads to low latency. In contrast, the centralized cloud computing model transmits enormous data to the cloud server for computation, thus increasing traffic congestion and latency rate.

### 4.3. Limitations and Future Directions

The system proposed in this work deploys a fuzzy inference system for pre-processing combined with a modified GRU for heart attack risk prediction that outdoes other models, with remarkable outcomes evident from the performance results. The research initiative of performing health analytics at the fog layer has shown substantial results in reducing latency. However, the model harnesses the UCI heart disease dataset, and in order to ascertain the efficacy of the model, it needs to be deployed in a medical setting. Despite having significant upsides to the traditional cloud-only model, the fog model encounters several downsides that must be remedied. Fog computing encompasses a diverse set of end devices ranging among sensors, mobile phones, and internet-of-things gadgets, among others. The billions of geographically dispersed fog devices raise maintainability issues and increase operational expenses. In addition, it encounters the strenuous task of linking heterogeneous devices with different configurations that operate with multiple protocols and are furnished by multiple vendors. The seamless integration of such diverse devices and systems raises compatibility and interoperability issues. Hence, rigorous research efforts are needed to overcome these challenges.

Furthermore, because data is processed and stored on multiple edge devices, there is an increased risk of data breaches and unauthorized access with fog computing. This can be overcome by configuring the fog nodes to be impervious to physical harm and site-based attacks, with strict access control policies and tamper-proof hardware. Authentication and trust methods developed before the advent of heterogeneous IoT nodes and fog nodes are becoming obsolete. Hence, a novel user, service, node authentication, and trust framework must be designed. Offloading work to fog nodes could compromise personal data, so a foolproof method of offloading tasks while ensuring their correctness and integrity is needed. The user has access to multiple potentially sensitive fog nodes. Protecting the confidentiality of private information with appropriate privacy-preserving mechanisms is crucial.

## 5. Conclusions

The healthcare industry has primarily benefited from digitizing hospital records, enabling clinicians to leverage powerful predictive algorithms to facilitate clinical decision-making. It enables the prevention and cure of health issues by alerting clinicians, as well as caregivers, ahead of time on the possibility of medical events. In this research endeavour, a smart system for the prediction of heart attack risk based on an IoT–fog–cloud model is propounded, which deploys the fuzzy inference system (FIS) and a modified gated recurrent unit (GRU) to handle the predictive task. This suggested system outperforms other cutting-edge heart disease prediction models. The time-sensitive analytical tasks effectuated at the fog layer effectively tackle the IoT data upsurge, as they prevail over the cloud’s inherent shortcoming of higher latency caused by increased response time. The proffered model can be extended to facilitate personalized diet and exercise recommendations to patients on the clinician’s advice. Healthcare analytics expedites the early identification of illnesses, agile clinical decision-making, and timely intervention, enabling more accurate, swifter, and safer patient care. It further enhances the quality of service by involving medical data analytics at the fog layer. This is just a small fraction of the immense potential that deep learning models possess, which has yet to be explored in healthcare. The future of healthcare is anticipated to forge ahead with unfolding technology trends. Moreover, the complex challenges of device heterogeneity and geographically wide distribution, interoperability, maintainability, increased operational expenditure, and security and privacy concerns in the fog-based healthcare environment are to be resolved with further study.

## Figures and Tables

**Table 1:** Attribute description.

No.	Attribute	Description	Value Range
1	age	Patient age in years	29–77
2	sex	Gender instance	1 = Male; 0 = Female
3	cp	Type of chest pain	1 = Angina, 2 = Atypical form of angina, 3 = Non-angina, 4 = No symptoms of angina
4	trestbps	Resting blood pressure in mm Hg	[94; 200]
5	chol	Cholesterol value in mg/dL	[126; 564]
6	fbs	Fasting blood sugar Value > 120 mg/dL	1 = True and 0 = False
7	restecg	Value of ECG at rest	0 = Normal, 1 = Abnormal (ST-T wave), 2 = Definite Ventricular
8	thalach	Maximum heart rate recorded	[71; 202]
9	exang	Exercise induced angina	1 = yes; 0 = no
10	oldpeak	Exercise induced ST depression	[0.0; 62.0]
11	slope	slope of T segment peak exercise	1 = up-sloping, 2 = flat and 3 = down sloping
12	ca	Major vessels number coloured by fluoroscopy	0–3
13	thal	Defect types	3 = normal; 6 = fixed defect; 7 = reversable defect

**Table 2:** Dataset sample.

age	sex	cp	trestbps	chol	fbs	restecg	thalac	exang	oldpeak	slope	ca	thal
40	1	1	97	539	1	2	198	0	0.2	2	1	2
65	0	0	157	281	1	2	142	0	2	1	4	3
55	0	1	180	408	0	2	119	0	3	0	0	1
70	1	3	118	208	1	2	153	0	4	1	0	0
40	0	3	153	409	0	1	186	1	2.4	0	2	0
32	0	2	127	245	1	2	192	0	1.8	1	4	3
70	0	1	142	160	0	2	188	0	0.6	0	2	1
59	1	3	157	481	0	1	117	1	0.9	2	3	1
62	1	2	151	490	1	1	146	0	1.2	0	4	3
63	0	0	129	505	1	0	189	1	1	1	1	3

**Table 3:** Hyperparameter values—proposed model.

Hyperparameter	Value
Lag (Length of time lags)	1039
Number of layers	4
Number of hidden layers	2
Number of units in dense layer	5
Dropout rate	16
Decay rate	0.94
Learning rate	0.018
Number of epochs	765
Batch size	128

**Table 4:** Comparing performance measures of the proposed system.

Performance Metrics	GRU	FGRU	Proposed
Accuracy (%)	96.55	98.85	99.1250
Precision (%)	96.57	98.90	99.1300
Recall (%)	96.53	98.81	99.1200
Specificity (%)	96.57	98.90	99.1299
F1 Score (%)	96.55	98.86	99.1250

**Table 5:** Comparison with state-of-the-art systems.

No.	Author/Year	Method	Accuracy (%)
1	Desai et al. [26], 2022	Logistic Regression	85.96%
2	Singh et al. [16], 2020	K-Nearest Neighbor	87.00%
3	Subhahi et al. [29], 2022	Kernel Discriminant Analysis and Modified Self-Adaptive Bayesian Algorithm	90.00%
4	Rajdhan et al. [17], 2020	Random Forest	90.16%
5	Liu et al. [19], 2017	Relief and Rough Set Feature Selection and Ensemble Classifier Model	92.59%
6	Khan et al. [28], 2020	Modified Deep Convolutional Neural Network	93.30%
7	Nancy et al. [4], 2022	Fuzzy Inference System and Modified Bi-LSTM	97.32%
8	Nashif et al. [18], 2018	Support Vector Machine	97.53%
9	Rao et al. [22], 2021	Ensemble Deep Dynamic Algorithm	98.12%
10	Liu et al. [20], 2018	Symbolic aggregate Approximation and Long Short-Term Memory	98.40%
11	Proposed Model	Fuzzy Inference System and Gated Recurrent Unit	99.13%

## Data Availability

The datasets generated and/or analysed during the current study are available in the UCI machine learning repository, URL: https://archive.ics.uci.edu/dataset/45/heart+disease (accessed on 12 June 2023). The original contributions generated for this study are included in the article; further inquiries can be directed to the corresponding author.

## Abbreviations

IoT	Internet of Things
WHO	World Health Organization
ICU	Intensive Care Unit
FC	Fog Computing
AI	Artificial Intelligence
ML	Machine Learning
DL	Deep Learning
QoS	Quality of Service
RNN	Recurrent Neural Network
CNN	Convolutional Neural Network
ANN	Artificial Neural Network
GRU	Gated Recurrent Unit
ReLU	Rectified Linear Unit
EHR	Electronic health record
UCI	University of California Irvine
ECG	Electrocardiogram
EEG	Electroencephalogram
EMG	Electromyogram
LSTM	Long Short-Term Memory
FIS	Fuzzy Inference System
IDC	International Data Corporation
ZB	Zettabytes
ROC	Receiver Operating Characteristics
AUC	Area Under Curve
mAP	Mean Average Precision
